# Micro/nano-textured hierarchical titanium topography promotes exosome biogenesis and secretion to improve osseointegration

**DOI:** 10.1186/s12951-021-00826-3

**Published:** 2021-03-19

**Authors:** Zhengchuan Zhang, Ruogu Xu, Yang Yang, Chaoan Liang, Xiaolin Yu, Yun Liu, Tianlu Wang, Yi Yu, Feilong Deng

**Affiliations:** 1 Department of Oral Implantology, Hospital of Stomatology, Guanghua School of Stomatology, Sun Yat-Sen University, No.56 of LingYuanXiLu, Guangzhou, 510055 Guangdong People’s Republic of China; 2 Guangdong Provincial Key Laboratory of Stomatology, Guangzhou, People’s Republic of China

**Keywords:** Micro/nanonet-textured hierarchical titanium topography, Micro/nanotube-textured hierarchical titanium topography, Bone marrow mesenchymal stem cells, Exosomes, Osseointegration

## Abstract

**Background:**

Micro/nano-textured hierarchical titanium topography is more bioactive and biomimetic than smooth, micro-textured or nano-textured titanium topographies. Bone marrow mesenchymal stem cells (BMSCs) and exosomes derived from BMSCs play important roles in the osseointegration of titanium implants, but the effects and mechanisms of titanium topography on BMSCs-derived exosome secretion are still unclear. This study determined whether the secretion behavior of exosomes derived from BMSCs is differently affected by different titanium topographies both in vitro and in vivo.

**Results:**

We found that both micro/nanonet-textured hierarchical titanium topography and micro/nanotube-textured hierarchical titanium topography showed favorable roughness and hydrophilicity. These two micro/nano-textured hierarchical titanium topographies enhanced the spreading areas of BMSCs on the titanium surface with stronger promotion of BMSCs proliferation in vitro. Compared to micro-textured titanium topography, micro/nano-textured hierarchical titanium topography significantly enhanced osseointegration in vivo and promoted BMSCs to synthesize and transport exosomes and then release these exosomes into the extracellular environment both in vitro and in vivo. Moreover, micro/nanonet-textured hierarchical titanium topography promoted exosome secretion by upregulating RAB27B and SMPD3 gene expression and micro/nanotube-textured hierarchical titanium topography promoted exosome secretion due to the strongest enhancement in cell proliferation.

**Conclusions:**

These findings provide evidence that micro/nano-textured hierarchical titanium topography promotes exosome biogenesis and extracellular secretion for enhanced osseointegration. Our findings also highlight that the optimized titanium topography can increase exosome secretion from BMSCs, which may promote osseointegration of titanium implants.

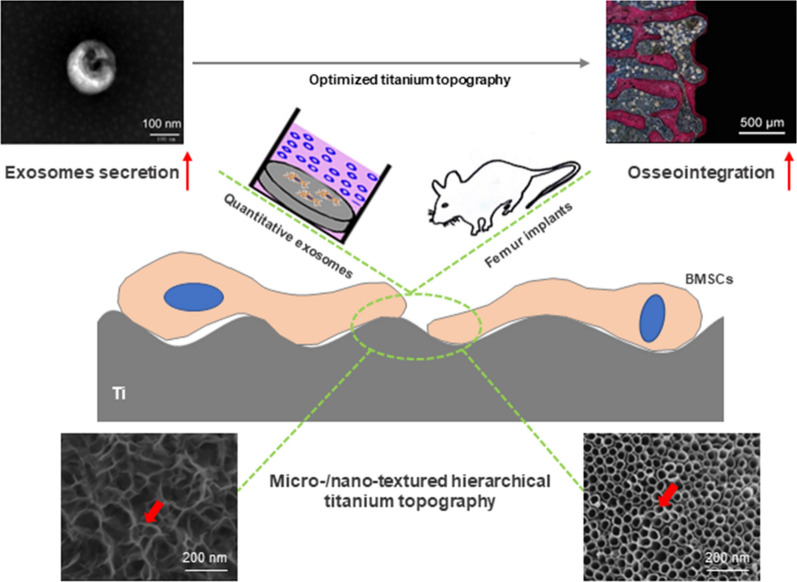

## Background

Dental implants in patients with osteoporosis have a failure rate of over 10% due to decreased alveolar bone density and poor regeneration ability after tooth loss [1, 2]. The proliferation and osteogenic differentiation capacities of bone marrow mesenchymal stem cells (BMSCs) in osteoporotic patients are inhibited [3]. BMSCs and secretome products derived from BMSCs play important roles in osseointegration between bone and titanium implants [4, 5]. The surface design of titanium implants has been highlighted to optimize a favorable titanium topography on the biological responses of BMSCs [6, 7]. However, much less is known about the role of titanium topography in the regulation of BMSCs-derived secretome products.

Exosomes, which are synthesized and secreted by various cell types as extracellular vesicles (EVs) [8], convey nucleic acids, proteins, lipids, and other active molecules into neighboring cells for cell-to-cell communication [9, 10]. The interactions among titanium implants, BMSCs, and exosomes are vital in the process of osseointegration [5]. It was shown in vivo and in vitro that exosomes secreted by BMSCs enhance adhesion, proliferation, and osteogenic differentiation of BMSCs and facilitate improved bone regeneration with a high concentration of exosomes by activating the PI3k/Akt pathway [11–13]. The small GTPase Rab27 and the sphingomyelin phosphodiesterase 3 (SMPD3) pathway control the synthesis and secretion of exosomes [14, 15]. The release of exosomes was notably reduced in Rab27-knockdown and SMPD3-inhibited cells using shRNA and GW4869 respectively [16, 17]. Recently, exosomes-integrated titanium disks were constructed with favorable biofunctionality for targeted osteogenesis by enhancing the adhesion, proliferation, and osteogenic differentiation of BMSCs [5, 18]. The physicochemical characteristics of titanium surfaces have been reported to affect the biological behavior and function of stem cells [19–21]. However, how titanium topography influences the secretion of exosomes from BMSCs is not clear.

Recently, customized titanium implants in intricately edentulous ridges have become possible with the rapid developments in the latest additive manufacturing (Selective Laser Melting, SLM), allowing for precisely controlled implant design [22, 23]. Moreover, the interface behavior between bone and SLM implants has been shown to be superior to the turned implants but inferior to that of commercial implants due to the excessive surface roughness of the SLM implants [24]. Surface modifications of turned or native-SLM titanium topography have been widely studied for improved performance of BMSCs on dental implants by constructing biomimetic topographies similar to the microstructure of bone tissue [25, 26]. Hierarchical porous structures have been widely studied as a nanocarrier for gene delivery and a framework for tissue regeneration [27–29]. Micro/nano-textured hierarchical titanium topographies have been reported to be more bioactive and biomimetic than smooth, native-SLM or micro-textured titanium topographies for enhanced osteogenic differentiation of BMSCs and bone-implant osseointegration in vitro and in vivo [30, 31]. Surface modifications of micro/nano-textured hierarchical titanium topographies usually occur through anodic oxidation, alkali-heat treatment, or electrochemical deposition on the micro-textured titanium base by sandblasting or acid etching [30, 32, 33]. In vitro studies have shown that micro/nano-textured topographies with combined acid etching and anodic oxidation techniques or combined acid etching and alkali-heat treatment techniques significantly enhance BMSCs behavior on the titanium surface [34, 35]. These studies focus only on the proliferation and osteogenic differentiation results of BMSCs under the designed topography. The precise mechanisms of micro/nano-textured topography-cell interactions for the enhanced BMSCs bioactivity and osseointegration are not known. Considering the importance of BMSCs-derived exosomes in osseointegration, micro/nano-textured topography may influence exosome secretion from BMSCs and then regulate cell-titanium activity and cell–cell communication on the titanium surface.

In this study, we determined whether the secretion behavior of exosomes derived from BMSCs is differently affected by different titanium topographies. Furthermore, we sought to explore the effect and mechanism of titanium topography on BMSCs-derived exosome secretion both in vivo and in vitro.

## Results

### Surface characterization

**Fig. 1:**
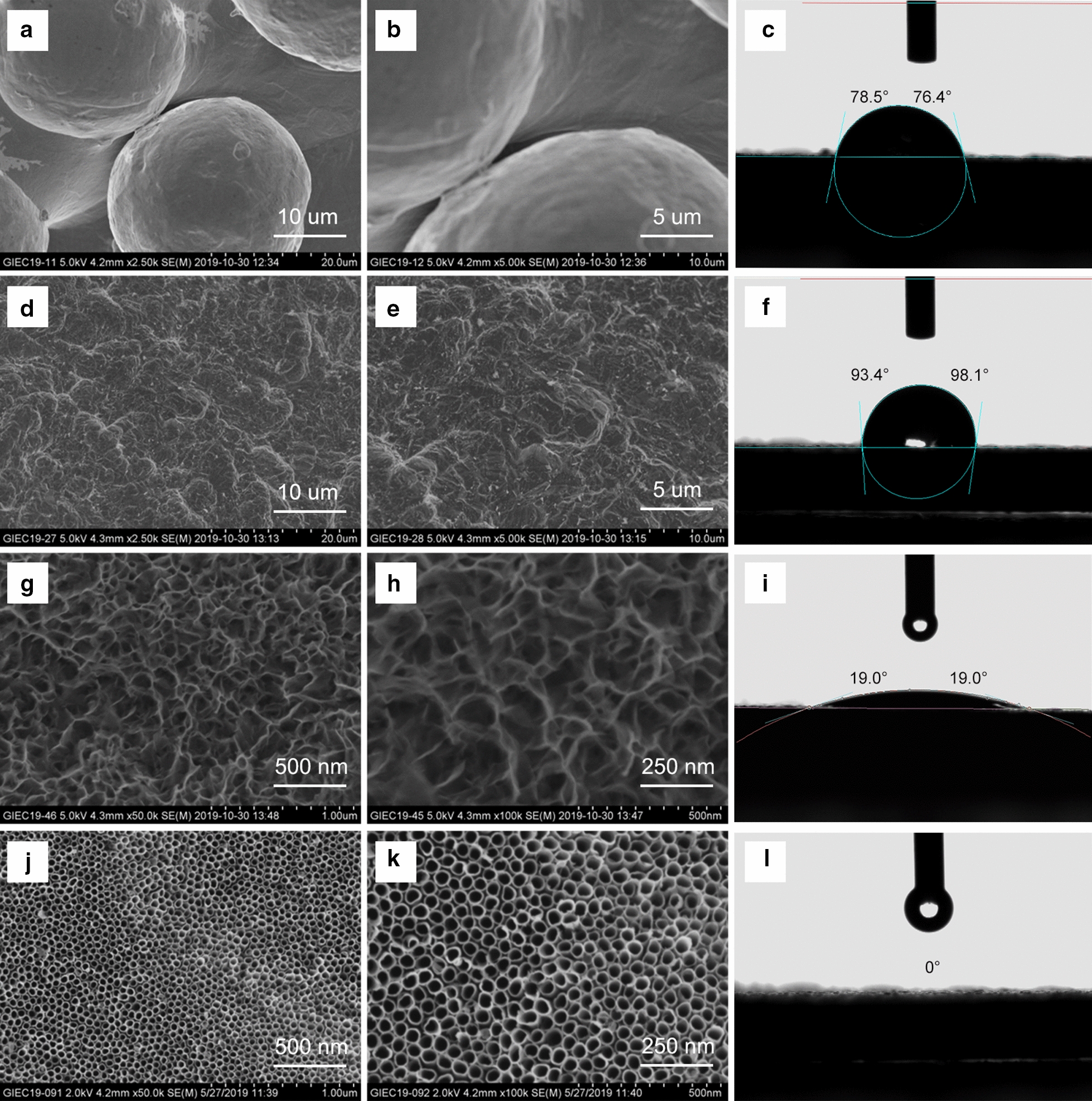
SEM images and water contact angles of the SLM, SLA, SAH, and SAO samples. SEM images of the SLM group at 2500× magnification (**a**) and at 5000× magnification (**b**). Water contact angle of 77.5 ± 1.1° for the SLM group (**c**). SEM images of the SLA group at 2500× magnification (**d**) and at 5000× magnification (**e**). Water contact angle of 95.8 ± 2.4° for the SLA group (**f**). SEM images of the SAH group at 50,000× magnification (**g**) and at 100,000× magnification (**h**). Water contact angle of 19.0 ± 0° for the SAH group (**i**). SEM images of the SAO group at 50,000× magnification (**j**) and at 100,000× magnification (**k**). Water contact angle of 0 ± 0° for the SAO group (**l**)

Field-emission scanning electron microscopy (FE-SEM) was used to determine the surface topographies of each group. The SLM samples showed rough and waved surfaces with some unmelted titanium spheres (diameter 28.29 ± 0.64 μm) (Fig. 1a, b). The SLA samples displayed well-distributed micrometer-sized pits (average diameter 5.62 ± 0.65 μm) without residual particles from the SLM samples after sandblasting and acid etching (Fig. 1d, e). Nano-textured titanium topography was not found on the surfaces of either the SLM or SLA samples. At low magnification, the SAH and SAO samples showed the same shallow pitted surfaces over 1 μm in diameter similar to the micrometer-sized pits on the SLA surface (Fig. 1g, j). At high magnification, bionic structures with interpenetrating hierarchical nanonets were observed on the SAH samples with 301.52 ± 51.22 nm large pores outside and 76.27 ± 15.57 nm small pores inside (Fig. 1h). Uniformly arranged nanotubes (average diameter 55.36 ± 6.16 nm) were formed on the SAO samples (Fig. 1k).

The surface roughness results (Sa and Sq) are shown in Table 1. The SLM samples exhibited significantly higher Sa and Sq values than those of the SLA (*p* < 0.05), SAH (*p* < 0.05), and SAO samples (*p* < 0.05). There were no significant differences among the SLA, SAH, and, SAO surfaces (*p* > 0.05).

**Table 1:** Surface roughness results of each titanium topography (n = 3)

Sample	Sa (μm)	Sq (μm)
SLM	13.05 ± 0.25^bcd^	16.51 ± 0.20^bcd^
SLA	4.21 ± 0.57^a^	5.07 ± 0.47^a^
SAH	4.19 ± 0.21^a^	5.24 ± 0.26^a^
SAO	4.57 ± 0.17^a^	5.89 ± 0.51^a^

a, b, c, and d indicate significant difference compared to SLM, SLA, SAH, and SAO respectively (*p* < 0.05)

The water contact angles of the SLM, SLA, SAH, and SAO samples were 77.5 ± 1.1°, 95.8 ± 2.4°, 19.0 ± 0°, and 0 ± 0°, respectively (Fig. 1c, f, i, and l). The SAH surface showed favorable hydrophilicity, while the SAO surfaces displayed superhydrophilicity.

### Identification of hBMSCs

**Fig. 2:**
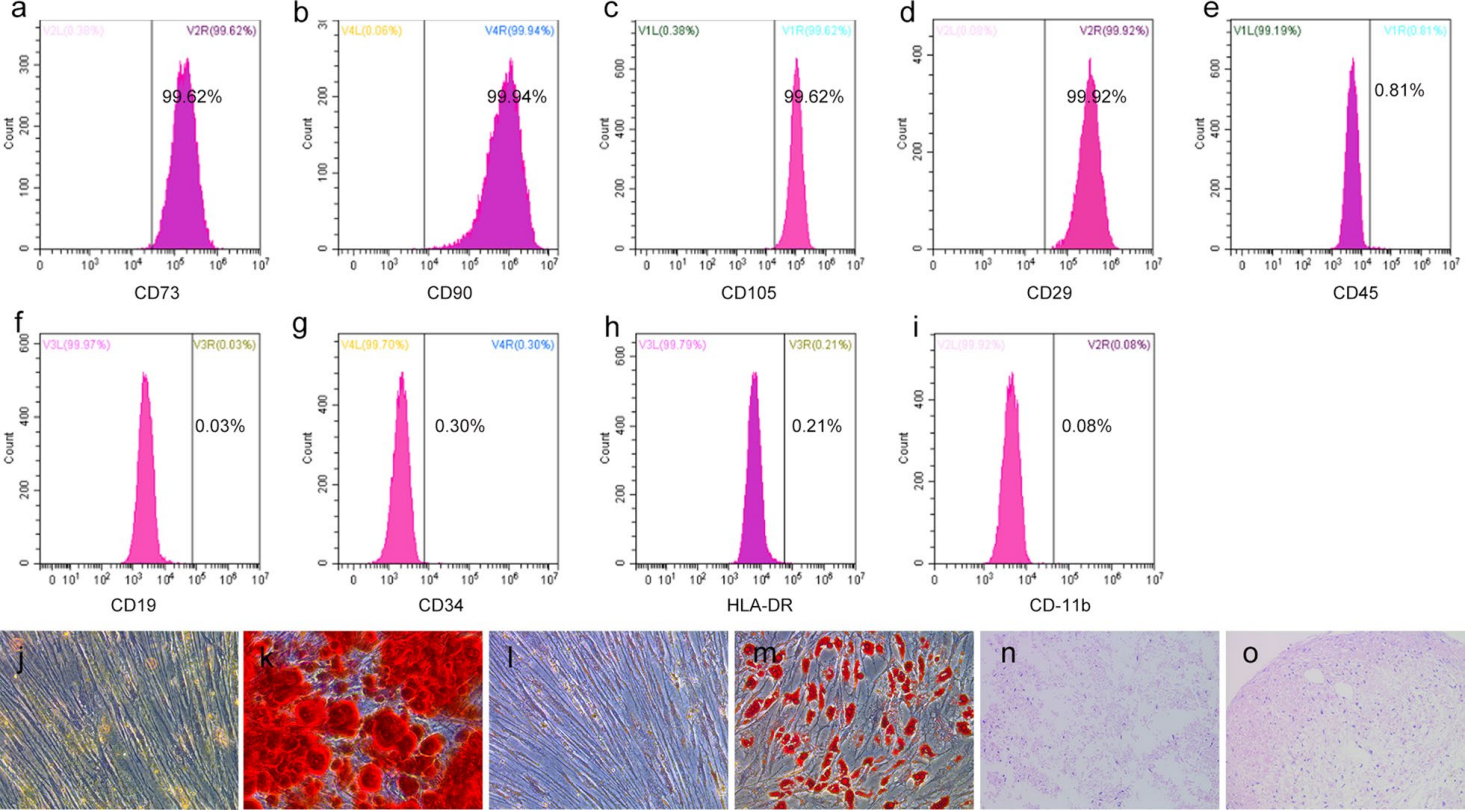
The immunophenotypic and differentiation potential results of hBMSCs were determined. Surface antigen markers results of mesenchymal stem cells (**a**–**d**). Surface antigen markers results of hemopoietic cells (**e**–**i**). The staining results for non-induction, osteogenic, lipogenic and chondrogenic induced differentiation of hBMSCs at 200× magnification by Alizarin red staining of non-induction (**j**) and osteogenic induction (**k**), oil red O staining of non-induction (**l**) and lipogenic induction (**m**), and safranin O staining of non-induction (**n**) and chondrogenic induction (**o**)

Surface marker expression was confirmed using flow cytometry. hBMSCs showed an over 99% positive rate for mesenchymal stem cell markers (CD73, CD90, CD105, and CD29) and the absence of hematopoietic surface antigens (CD45, CD19, CD34, HLA-DR, and CD11b) (Fig. 2a–i).

The osteogenic, adipogenic, and chondrogenic differentiation potential of the hBMSCs was determined compared to the noninduced group. After 11 days under osteogenic differentiation culture conditions, the cells showed extracellular calcium depositions by Alizarin red staining (Fig. 2j, k). After 11 days under adipogenic differentiation culture conditions, the cells showed lipid inclusion formation by oil red O staining (Fig. 2l, m). After 21 days under the chondrogenic differentiation culture conditions, the slices of cell conglomerates displayed proteoglycan depositions and cartilage lacuna formation by safranin O staining (Fig. 2n, o).

### Cell viability evaluation

**Fig. 3:**
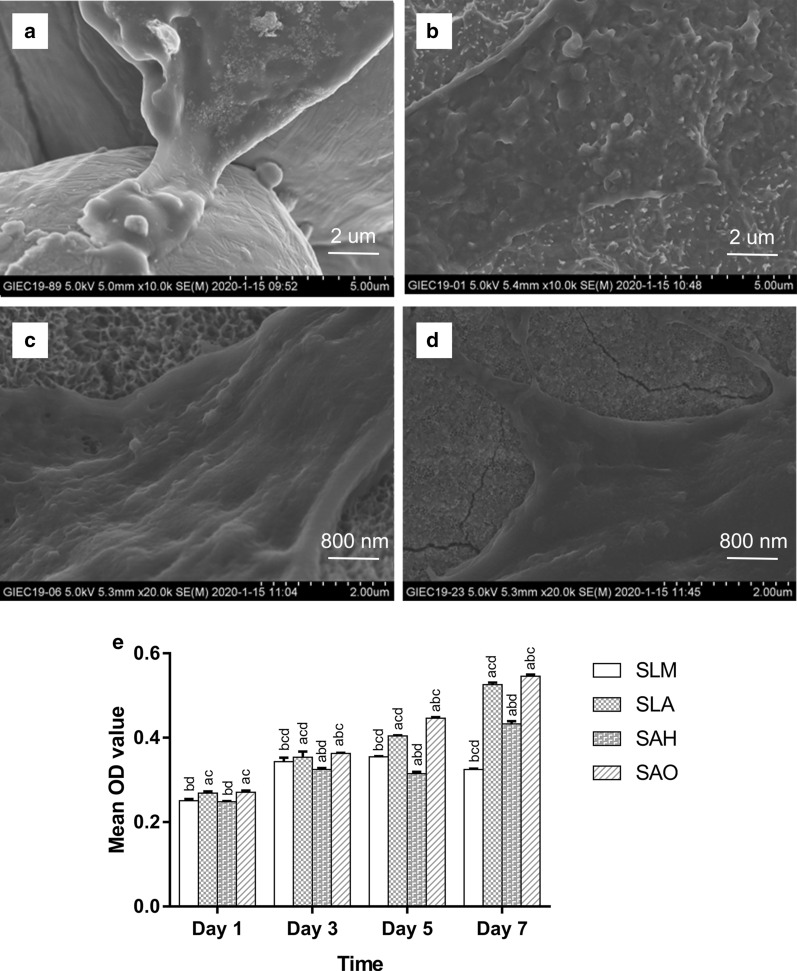
The cell adhesion, morphology, and proliferation of hBMSCs on the titanium surface of each group. SEM images of BMSCs on the SLM group at 10,000× magnification (**a**), SLA group at 10,000× magnification (**b**), SAH group at 10,000× magnification (**c**), and SAO group at 10,000× magnification (**d**). Cell proliferation evaluation of hBMSCs on the titanium surface of each group after 1, 3, 5 and 7 days by CCK-8 (**e**) (n = 3). **a**–**d** above each bar indicate significant difference compared to SLM, SLA, SAH, SAO respectively (*p* < 0.05).

Cell adhesion and morphology were evaluated by SEM. Cells on the SLM and SLA samples were polygonal with short lamellipodia (Fig. 3a, b). Elongated cell bodies with finger-like filopodia protrusions were found in both the SAH and SAO samples and tiny protrusions were extended from these cells into the micro/nano-textured hierarchical titanium structure (Fig. 3c, d).

Cell proliferation was evaluated by CCK-8 assay (Fig. 3e). On the first day, there were no significant differences between the SAO and SLA group (*p* > 0.05), or between the SAH and SLM group (*p* > 0.05). The SAO group with micro/nanotube-textured hierarchical titanium topography had the strongest promotion of hBMSCs proliferation on the third, fifth, and seventh days (*p* < 0.05), followed by the SLA group with micro-textured titanium topography and the SAH group with micro/nanonet-textured hierarchical titanium topography.

### Characterization of the hBMSCs-derived exosomes

**Fig. 4:**
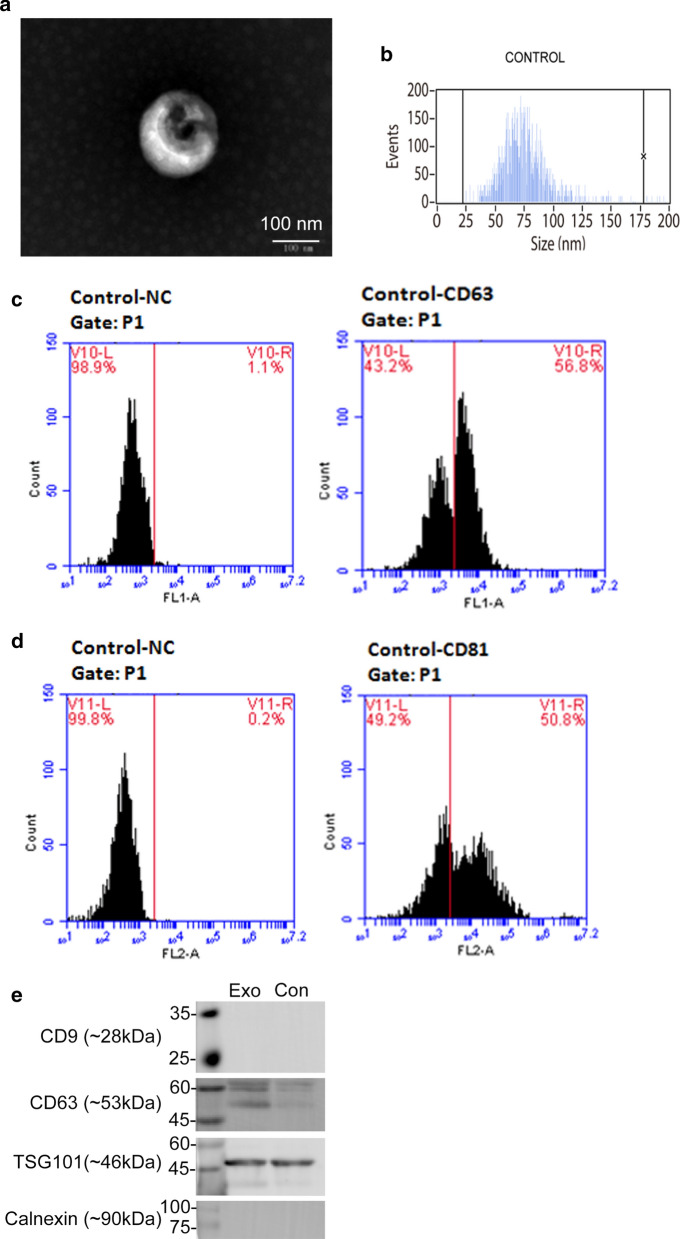
Characterization of hBMSCs derived exosomes. Transmission electron micrograph image of exosomes (**a**). Particle size distribution with the size range of exosomes by Flow NanoAnalyzer (**b**). CD63 surface antigen marker of exosomes by flow cytometry (**c**). CD81 surface antigen marker of exosomes by flow cytometry (**d**). Representative western blots showing exosome markers (CD9, CD63 and TSG101) and an intracellular marker (Calnexin) of the exosome sample (Exo) and controlled culture medium with 10% exosome-depleted FBS (Con) (**e**)

The hBMSCs-derived exosomes showed monolayer membrane vesicles with round grape-like structures (Fig. 4a). The particle size distribution of the samples was 30–150 nm (median ± SD, 73.0 ± 18.7 nm) in diameter by nano-flow cytometry (Fig. 4b). Flow cytometry analysis of the samples demonstrated positive expression of both CD63 (56.8%) and CD81 (50.8%), which are markers of exosomes (Fig. 4c, d). Moreover, western blotting analysis showed that the hBMSCs-derived exosomes demonstrated positive expression for exosome markers (CD63, and TSG101) and the absence of endoplasmic reticulum antigen (Calnexin) (Fig. 4e).

### Effects of titanium topography on exosome secretion via AChE activity

**Fig. 5:**
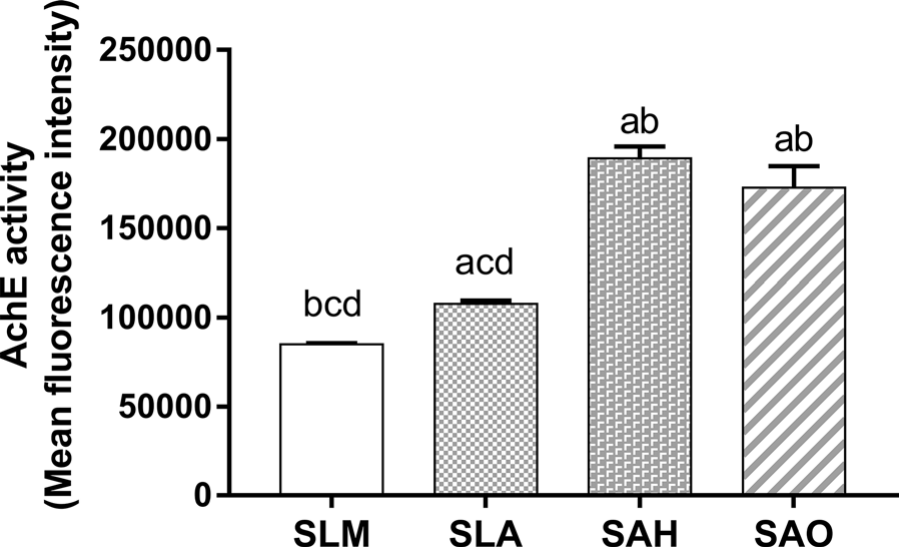
Topography effect of titanium surface on AChE activity of hBMSCs derived exosomes (n = 3). The relative numbers of the exosomes in the supernatant from hBMSCs on the SLM, SLA, SAH, and SAO samples were determined by the fluorescence values of the exosome samples at 590 nm emission detection. **a**–**d** above each bar indicate significant difference compared to SLM, SLA, SAH, SAO respectively (*p* < 0.05)

Exosomes released into the supernatant were quantitatively measured via the AChE activity of the isolated exosomes (Fig. 5). The activities of acetylcholinesterase were significantly higher in the SAH and SAO groups than in the SLM and SLA groups (*p* < 0.05). Moreover, the SLA group secreted significantly more exosomes than the SLM group (*p* < 0.05).

### Intracellular CD63 distribution on different titanium topographies

**Fig. 6:**
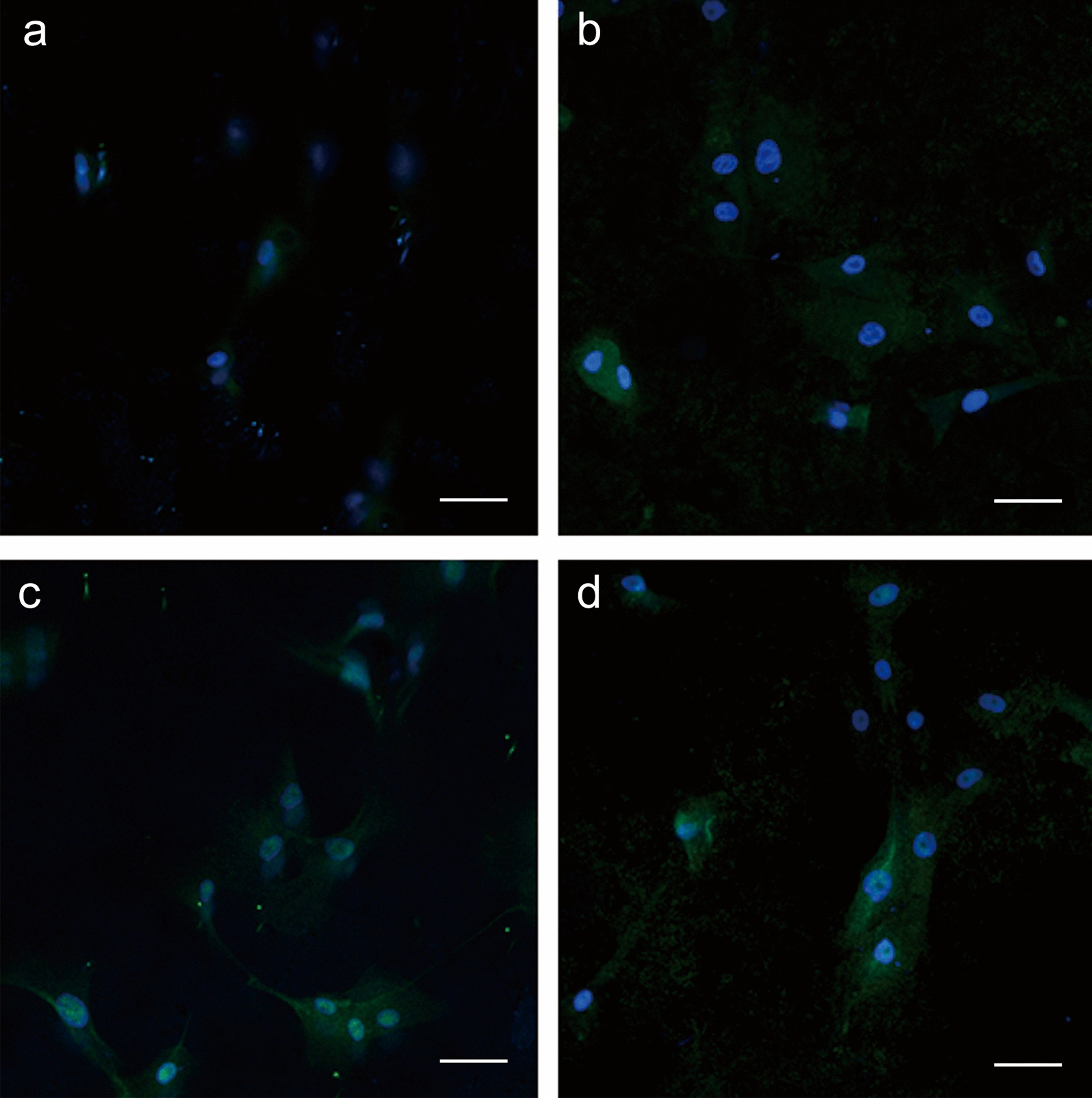
The adhesion morphology, nucleus, content, and distribution of intracellular CD63 of hBMSCs on each sample after co-culture for 48 h at 20× magnification. Confocal laser scanning microscope images of the SLM (**a**), SLA (**b**), SAH (**c**), and SAO (**d**) group (blue fluorescence, nucleus staining with DAPI; green fluorescence, CD63 staining with Alexa Fluor 488; scale bar = 50 μm)

Immunofluorescent staining results revealed similar cell nuclei of the four groups, and similar adhesion and morphology results were observed in the cell viability evaluation. Cells on the SLA samples (Fig. 6b) showed more spreading areas and intracellular CD63 distribution than those on the SLM samples (Fig. 6a). Cells in the SAH (Fig. 6c) and SAO (Fig. 6d) groups with elongated cell bodies connected to neighboring cells had more intracellular CD63 distribution than cells in the SLM and SLA groups.

### Effects of titanium topography on gene and protein expression levels related to exosome secretion

**Fig. 7:**
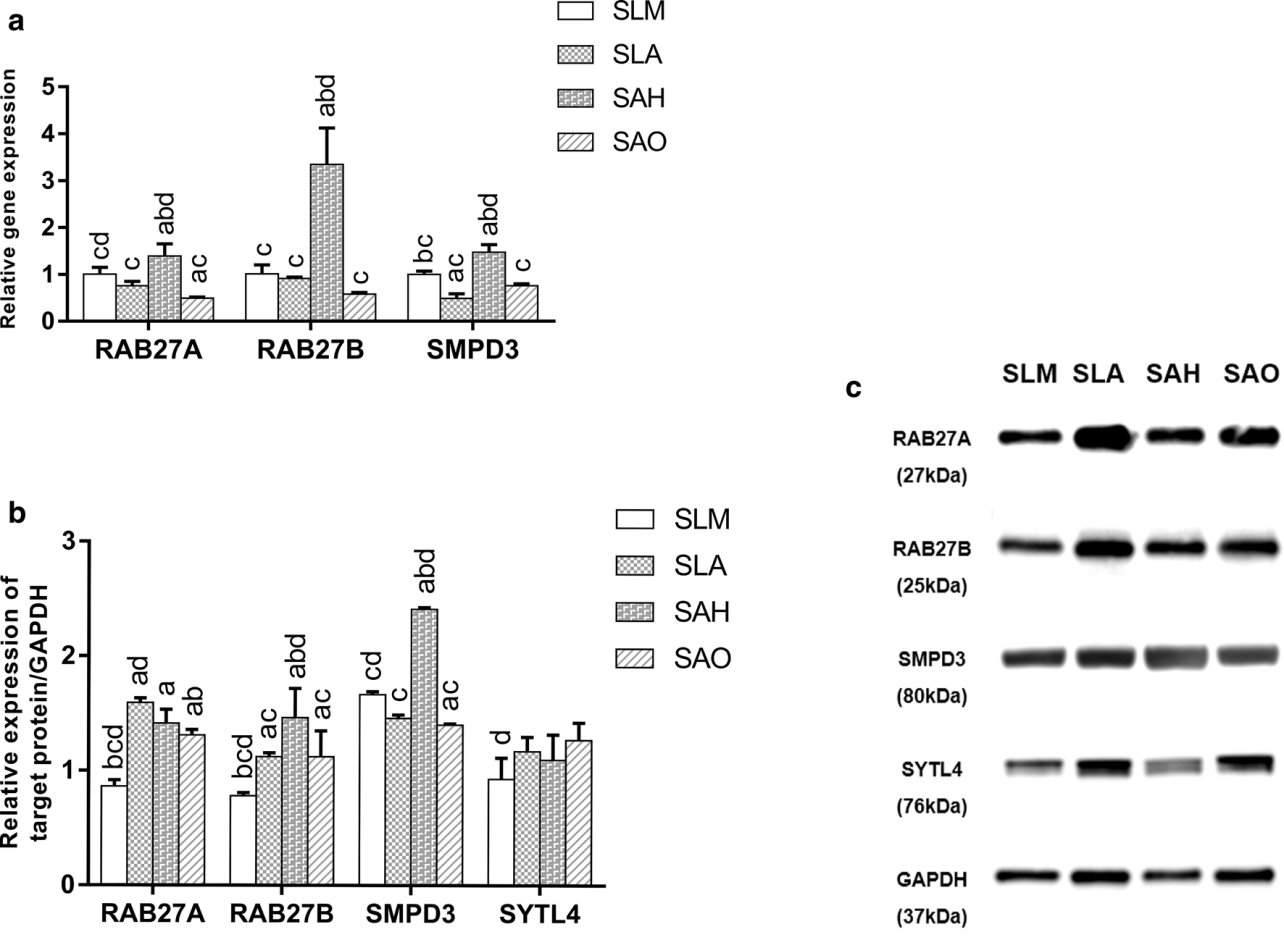
Effects of titanium topography on gene and protein expression levels related to exosome secretion. Gene expression evaluation related to exosome secretion of hBMSCs on the titanium surface of each group by RT-qPCR (**a**) (n = 3). Protein expression evaluation related to exosome secretion of hBMSCs on the titanium surface of each group by quantitative analysis (**b**) of Western blotting bands (**c**) (n = 3). a–d above each bar indicate significant difference compared to SLM, SLA, SAH, SAO respectively (*p* < 0.05)

Previous reports have shown that the Rab27 family and SMPD3 signaling control exosome secretion in a series of steps. After culturing for 48 h, the relative mRNA expression levels were determined by qRT-PCR (Fig. 7a). The micro/nanonet-textured hierarchical titanium topography significantly increased the mRNA levels of RAB27A, RAB27B, and SMPD3 compared to the other three groups (*p* < 0.05), but there was no significant difference in the mRNA levels between the SAO and SLA groups (*p* > 0.05). After culturing for 48 h, the relative protein expression levels were determined by WB (Fig. 7b, c). Micro/nanonet-textured hierarchical titanium topography significantly increased the protein abundance of RAB27B and SMPD3 compared to the other three groups (*p* < 0.05), but there was no significant difference in SYTL4 expression (a Rab27 effector) among the SAH, SAO, and SLA groups (*p* > 0.05). The SLM group showed significantly higher SMPD3 protein expression than the micro/nanotube-textured hierarchical titanium topography group (*p* < 0.05).

### Effects of titanium topography on exosome secretion and osseointegration in vivo

**Fig. 8:**
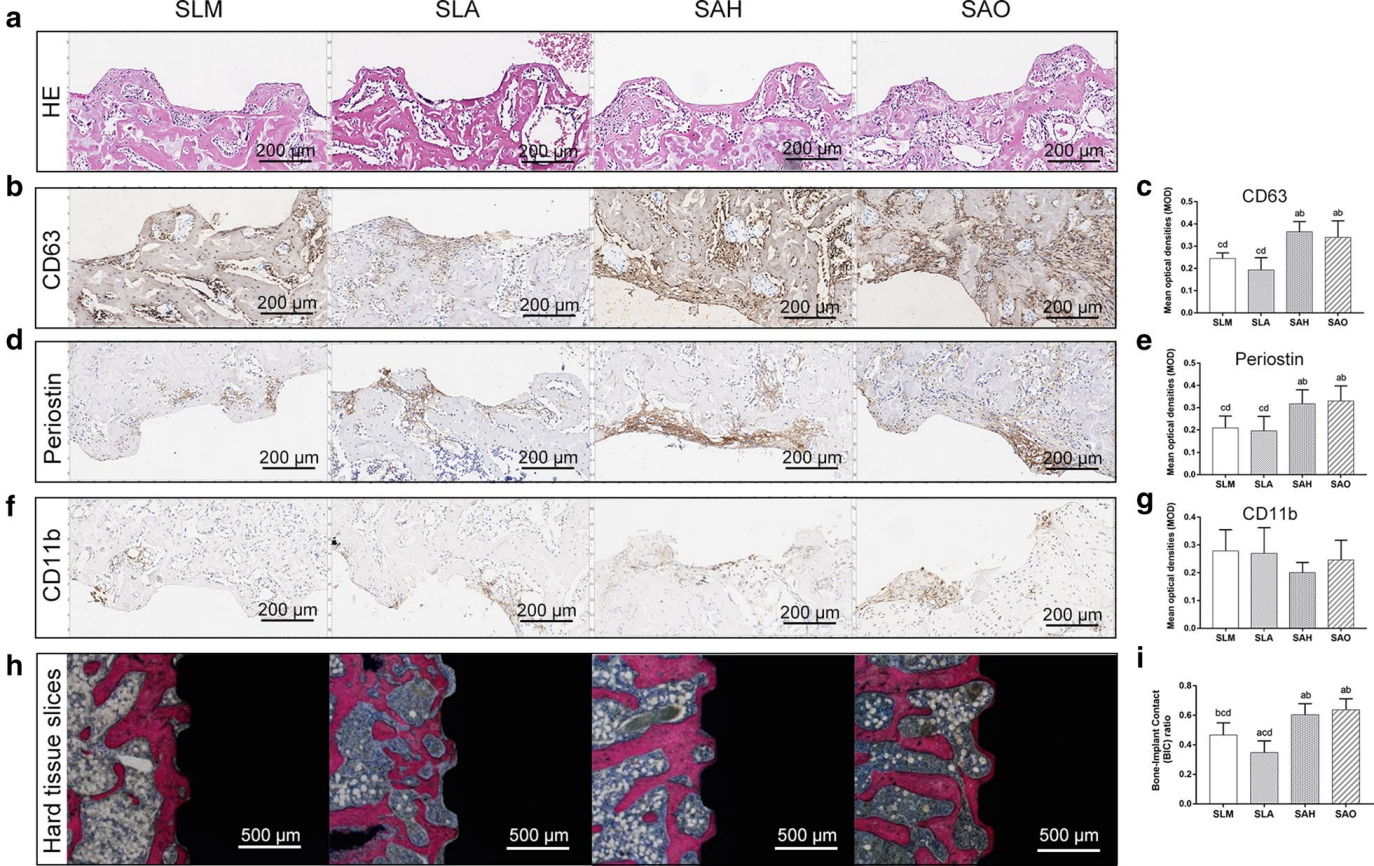
Effects of titanium topography on exosome secretion and osseointegration in vivo. Hematoxylin and eosin (H&E) staining of the bone-implant interface on the 7th day after surgery (**a**). The immunohistochemical assay results and the mean optical density (MOD) evaluation to show the expression levels of CD63 (a marker for exosomes) (**b**, **c**), periostin (a marker for mesenchymal stem cells) (**d**, **e**) and CD11b (a marker for tissue macrophages) (**f**, **g**) on the 7th day after surgery (n = 4). Histological analysis of the bone-implant contact (BIC) ratio (**h**, **i**) on the 28th day after surgery (n = 4). (**a**, **b**, **d** and **f**, scale bar = 200 μm; h, scale bar = 500 μm). **a**–**d** above each bar indicate significant difference compared to SLM, SLA, SAH, SAO respectively (*p* < 0.05)

On the 7th day after implant placement, H&E staining of the implant sites showed clear threads and coronal-to-apical aligned osteogenic cells. No infiltration superiorities of inflammatory cells in the bone-implant interface were protruding in any of the four groups (Fig. 8a). The exosome levels in the bone-implant interface were detected by a CD63 immunohistochemical assay. The expression levels of CD63 were significantly higher in the SAH and SAO groups than in the SLM and SLA groups (*p* < 0.05) (Fig. 8b, c). Periostin (a marker for mesenchymal stem cells)-positive cells were observed and quantified. The expression levels of periostin were significantly higher in the SAH and SAO groups than in the SLM and SLA groups (*p* < 0.05) (Fig. 8d, e), which was consistent with the exosome expression levels. No significant differences in the expression levels of CD11b (a marker for tissue macrophages) were found among the four groups (*p* > 0.05), but CD11b-positive cells gathered close to the bone-implant interface (Fig. 8f, g).

On the 28th day after implant placement, histological analysis revealed that the BIC ratios of the SAH and SAO groups were significantly higher than those of the SLM and SLA groups (*p* < 0.05) (Fig. 8h, i).

## Discussion

To our knowledge, this is the first observation that the micro/nano-textured titanium topography can influence exosome secretion from BMSCs in vitro and in vivo. In our experiments, we found that micro/nanonet-textured hierarchical titanium topography and micro/nanotube-textured hierarchical titanium topography showed favorable roughness and hydrophilicity. Micro/nano-textured hierarchical titanium topography enhanced the spreading areas of BMSCs on the titanium surface with a stronger promotion of BMSCs proliferation in vitro and osteointegration in vivo. Compared to micro-textured titanium topography, micro/nano-textured hierarchical titanium topography significantly promoted BMSCs to synthesize and transport exosomes and then release these exosomes into the extracellular environment. Moreover, micro/nanonet-textured hierarchical titanium topography promoted exosome secretion by upregulating RAB27B and SMPD3 gene expression and micro/nanotube-textured hierarchical titanium topography promoted exosome secretion due to the strongest enhancement in cell proliferation.

In our study, micro/nano-textured titanium topography was constructed based on the 3D-printed titanium disks using a selective laser melting technique with one-step molding, high precision, good mechanics, high digitization, customized design, and many other advantages [36, 37]. After sandblasting and acid etching, the unmelted titanium spheres were removed and well-distributed micrometer-sized pits were formed. There are many common acid etching processing methods to remove residual particles during the process of sandblasting by dissolving titanium oxide layers, such as the use of hydrochloric acid (HCl), hydrofluoric acid (HF), nitric acid (HNO_3_), sulfuric acid (H_2_SO_4_), and mixed acid solutions (HCl/H_2_SO_4_ or HF/H_2_SO_4_), but the titanium topography differs with the changes in acid concentration, treatment time and temperature [38–40]. We used a 5% HF solution treated for 2 min at room temperature and successfully constructed micro-textured titanium topography with superior time and operability [33, 41].

Nanoscale structures can improve the tissue response of osteoblasts, enhance osseointegration, reduce bacterial adhesion, and construct a drug delivery system [42]. Both the SAH and SAO samples showed shallow pitted surfaces with diameters of over 1 μm similar to the micrometer-sized pits on the SLA surface. After alkali-heat and anodic oxidation treatments, micro/nanonet-textured and micro/nanotube-textured hierarchical titanium topographies were formed, respectively. The micro/nanonet-textured hierarchical titanium topography with small and large nanoscale-sized pores is similar to the structure of the extracellular matrix (ECM) structure, which can provide the location and space in different directions for cell growth [43, 44]. Some studies have indicated that uniformly arranged titanium nanotubes with an average diameter of 70 nm possess favorable wettability, protein adsorption capacity, and antibacterial properties for improved osteogenesis [45–47]. In our study, an average nanotube diameter of 55.36 nm was produced, which should be beneficial for cell growth. A clear future challenge is optimization of the precise and repeatable micro/nano-textured hierarchical titanium topography.

A moderately rough (Sa 1–2 μm) and hydrophilic surface with different nanoscale structures can induce a predominant cellular response [48]. The roughness values are mainly influenced by the microscale structures, causing excessive surface roughness of the SLM samples due to the partially melted particles. Therefore, alkali-heat and anodic oxidation treatments for nanoscale structures could not decrease the roughness of the SLA samples, because the SLA, SAH, and SAO samples have similar microscale structures [49]. In contrast, the nanoscale structures are the determining factor of titanium hydrophilicity with a favorable microenvironment for osteogenesis and long-term maintenance of peri-implant bone level [50, 51].

The precise mechanisms for enhanced topography-related BMSCs spreading, adhesion, and proliferation are not known. In this study, micro/nano-textured titanium topography promoted the adhesion and proliferation of BMSCs on titanium surfaces. BMSCs tend to grow on an appropriate surface with a moderately rough and nano-textured topography, on which the finger-like filopodia protrusions extend into the nanonet or nanotube, leading to the formation of ECM and new bone [52, 53]. However, the SAH samples may not have clear cell proliferation superiority due to its highly spreading morphology with the restricted total area compared to the SLA samples with smaller cell bodies.

Exosomes secreted by BMSCs were extracted from the supernatant by an exosome isolation kit and identified in terms of morphology, particle size distribution, and antigen markers. Western blot and flow cytometry analysis showed that the extracted exosomes expressed CD63, CD81 and TSG101, which are commonly enriched in exosomes. The exosomes in this study showed high purity after measuring the positive rates of CD63 (56.8%) and CD81 (50.8%), similar to the gold isolation standard using the ultracentrifugation method with 46.0% and 55.0% positive percentages of CD63 and CD81 [54]. Based on quantitative results, micro/nano-textured hierarchical titanium topography significantly promoted BMSCs to synthesize and transport exosomes and then release these exosomes into the extracellular environment via AChE activity and intracellular CD63 evaluation, as described previously [15, 55]. The CD63 expression levels in the bone-implant interface complemented the findings in vitro.

The small GTPase Rab27 and the sphingomyelin phosphodiesterase 3 (SMPD3) pathway control the synthesis and secretion of exosomes [15, 56]. Our findings demonstrated that micro/nanonet-textured hierarchical titanium topography promoted exosome secretion by upregulating RAB27B and SMPD3 gene expression. However, SAO samples have no clear secretion superiority of exosome-related gene and protein expression due to the strongest enhancement in cell proliferation, which may exert very large numbers of cells to secrete exosomes. There were increased mesenchymal and osteoprogenitor positive cells around the titanium implants in vivo, as described previously [57], and therefore, the osteogenic effects of micro/nano-textured hierarchical titanium topography may be explained by increased numbers of BMSCs and stimulation of exosome secretion from BMSCs based on the in vitro results of the present study and other studies [5, 11]. Finally, we might regulate cell-titanium activity and cell–cell communication for better osseointegration by optimizing the titanium surface topography.

## Conclusions

In summary, it has been concluded that micro/nanonet-textured hierarchical titanium topography promotes exosome biogenesis and extracellular secretion by upregulating RAB27B and SMPD3 gene expression and micro/nanotube-textured hierarchical titanium topography promotes exosome secretion due to the strongest enhancement in cell proliferation for improved osseointegration. Our findings also highlight the important effects of titanium topography on regulating exosome release from BMSCs, but the detailed mechanism of the titanium-exosome-BMSCs interactions requires further study. Furthermore, the proteomic analysis of exosomal cargos from different topographies should be conducted to gain insight into the possible mechanism.

## Methods

### Specimen preparation and surface treatment

The titanium disks (diameter 10 mm; thickness 1 mm) and titanium implants (diameter 2.2 mm; length 5 mm) were designed by SolidWorks^®^ 12.0 software (SolidWorks Corp., USA) and manufactured by an SLM system (SLM125HL, SLM solutions GmbH, Germany) using commercially pure titanium powders (average 30 μm particle size; Grade II; Western BaoDe, PR China), as previously described [30, 33]. Briefly, the laser printing parameters of the SLM disks were set to 145 W power, 355 mm/s scanning velocity, 45 μm layer thickness, and 50 μm spot size. The SLM specimens were subsequently cleaned by compressed air and ultrasonic processing. These native titanium specimens were termed the SLM samples and used as the control group. Some of the SLM samples were sandblasted with 250 μm ZrO_2_ particles under constant 0.8 MPa compressed air for 10 s and then immersed in 5% hydrofluoric acid (HF) at room temperature for 2 min. These specimens were termed the SLA samples.

Some of the SLA samples were treated in a continuously stirring solution of 5M NaOH in a water bath at 80 ℃ for 8 h. These specimens were termed the SAH samples. Other SLA samples were incubated with 0.3 wt% ammonium fluoride (NH_4_F) in an ethylene glycol (C_2_H_6_O_2_) solution at 30 V for 60 min. These specimens were termed the SAO samples.

Four groups, the SLM, SLA, SAH, and SAO samples were ultrasonically cleaned and sterilized before use in each experiment.

### Surface characterization

The surface topography of each group was observed by field-emission scanning electron microscopy (FE-SEM; S-4800, Hitachi, Japan) and analyzed by ImageJ 1.52a software (National Institutes of Health, USA). Surface roughness parameters including arithmetic mean deviation roughness (Sa) and root mean square roughness (Sq) were determined using a confocal 3D surface profiler (UP-WLI, Rtec UP series, USA). Water contact angles of each group were detected using the sessile-drop method with a contact angle measuring system (OCA40 Micro, Dataphysics, Germany).

### Cell culture and identification assay

Human bone marrow mesenchymal stem cells (hBMSCs; Guangzhou Jennio Biotech Co., PR China) were cultured and expanded at 37 °C and 5% CO_2_ in basal growth DMEM (Gibco, USA) containing 10% fetal bovine serum (FBS; Gibco), 1% penicillin–streptomycin (Gibco) and 1% GlutaMAX™ supplement (Gibco). Cells were collected at passages 3–6 upon reaching approximately 80–90% confluence for the follow-up experiments.

For the surface marker expression assay, cells at passage 3 were first blocked with 3% bovine serum albumin (BSA; Beyotime Biotech, PR China) and stained for 30 min with the following conjugated antibodies (BioLegend, USA): anti-CD73-PE, anti-CD29-PE, anti-CD11b-PE, anti-CD105-FITC, anti-CD45-FITC, anti-CD90-APC, anti-CD34-APC, anti-CD19-PE/Cy7, anti-HLA-DR- PE/Cy7 and isotype-matched control antibodies. The cells were washed to remove unconjugated antibodies and identified for surface antigen markers by flow cytometry with a flow cytometer (CytoFLEX, Beckman, USA) and FlowJo X software (FlowJo, USA).

For the osteogenic differentiation assay, cells at passage 5 were seeded at a density of 2500 cells/mL in a 6-well plate. Upon reaching 60–70% confluence, the cell culture medium was replaced with osteogenic differentiation media consisting of basal growth DMEM, 0.1 μmol/L dexamethasone (Sigma, USA), 50 μmol/L ascorbic acid (Sigma) and 10 mmol/L β-sodium glycerophosphate (Sigma). Control cells were cultured at the same density in regular DMEM for 11 days. The osteogenic differentiation capacity of the cells was assessed by alizarin red staining and imaged at 200× magnification. For the adipogenic differentiation assay, cells at passage 5 were seeded at a density of 2500 cells/mL in a 6-well plate. When the cells reached 80–90% confluence, the cell culture medium was replaced with adipogenic differentiation media consisting of basal growth DMEM, 1 μmol/L dexamethasone, 10 mg/L insulin (Life, USA), 0.5 mmol/L 3-isobutyl-1-methylxanthine (Sigma) and 0.2 mmol/L indomethacin (Sigma). Control cells were cultured at the same density in regular DMEM for 11 days. The adipogenic differentiation capacity of the cells was assessed by oil red O staining and imaged at 200× magnification. For the chondrogenic differentiation assay, a suspension of 300,000 cells at passage 5 was centrifuged at 1500 r/min for 5 min to form a pellet in a 15 mL sterile tube and incubated overnight. The cell culture medium was replaced with chondrogenic differentiation media consisting of basal growth DMEM, 10 ng/mL TGF β-1 (Sigma), 50 mg/L ascorbic acid, 0.1 nmol/L dexamethasone, 50 mg/mL ITS (Sigma), 1 mmol/L sodium pyruvate (Sigma), and 5.35 μg/mg linoleic acid (Sigma). The control cell pellet was cultured at the same density in regular DMEM for 21 days. Sections (5 μm thickness) of the pellets were first stained with hematoxylin solution. The chondrogenic differentiation capacity of the cells was assessed by safranin O staining and imaged at 200× magnification.

### Cell viability evaluation

For the cell adhesion and morphology assay, hBMSCs were incubated on the specimen surfaces at a density of 10,000 cells/mL in 48-well plates for 24 h and subsequently fixed in 2.5% glutaraldehyde solution overnight at 4 ℃. After dehydration in a gradient ethanol series (50, 75, 90, 95, and 100%) and drying in air, the specimens were sprayed with gold and detected by FE-SEM.

For the cell proliferation assay, hBMSCs were cultured on the specimen surfaces at a density of 2500 cells/mL in 48-well plates for 1, 3, 5, and 7 days and evaluated using a cell counting kit-8 assay (CCK-8; Dojindo, Japan) according to the manufacturer’s instructions. Briefly, 500 μL of basal growth DMEM with 10% CCK-8 reagent was added to each well and incubated for 90 min. The supernatant absorbance at 450 nm of each group was measured at predetermined time points.

### Isolation and characterization of the exosomes

Cells in culture medium with 10% exosome-depleted FBS (BI, Israel) were seeded on the specimen surfaces at a density of 40,000 cells/mL in 48-well plates for 48 h. The hBMSCs-released exosomes were extracted by an exosome isolation kit (EIQ3, H-Wayen, PR China) according to the manufacturer's instructions. Briefly, supernatants were first centrifuged at 3000×*g* for 15 min to remove the cells and cell debris and then mixed with the isolation regent (at half the volume of the supernatant) at 4 ℃ overnight. After incubation, the exosomes in the mixture were precipitated by centrifugation at 3000×*g* for 60 min and resuspended in 1 mL of the supernatant from above. The resuspensions were centrifuged at 10,000×*g* for 10 min. Then, the pellets were resuspended in 200 μL of PBS and further purified by centrifugation at 10,000×*g* for 5 min. The supernatant contained the exosomes, and all steps were performed at 4 °C.

Transmission electron microscopy (TEM), nano-flow cytometry, flow cytometry and western blotting were used to identify the exosomes. Briefly, 10 μL of exosome preparations were placed onto a copper mesh and deposited for 3 min. The samples were first negatively stained with phosphotungstic acid, dried for 5 min, and then imaged by TEM (JEM-1200EX, Japan Electronics Co., Ltd, Japan) operating at 80–120 kV. For particle size analysis, the exosome preparations, standard silica particles, and blank controls (PBS) were detected with nano-flow cytometry (Flow NanoAnalyzer, NanoFCM, PR China) at a low feed pressure (≤ 1.0 kPa) under the same conditions (laser power and scattering channel attenuation coefficient). Standard working curves for the silica particles between the scattered light intensity and the particle size were established to calculate the particle size distribution of the samples. For phenotyping analysis, 100 μL of exosome preparations were stained for 30 min with FITC-conjugated antibodies (BD, USA), including mouse-anti-human CD63 and mouse-anti-human CD81, and ultracentrifuged (Optima L-100xp, Beckman, USA) at 100,000*g* for 20 min. The pellets were resuspended in PBS for flow cytometry (Accuri C6, BD, USA). Western blotting was conducted to examine exosome positive markers (CD9, CD63 and TSG101) and an exosome negative marker (Calnexin). The proteins from the exosome sample and culture medium with 10% exosome-depleted FBS (blank control) were extracted by RIPA lysis buffer and quantified by a BCA Protein Assay Kit (exosome sample: 0.72 μg/μL, control: 0.46 μg/μL). Equal amounts of proteins (20 μg) from each sample were tested according to the standard protocols of western blotting, which is described in the western blotting section of methods. The primary antibody information is as follows: rabbit anti-CD9 (1:1,000; SBI, USA), rabbit anti-CD63 (1:1,000; SBI), rabbit anti-TSG101 (1:1,000; SBI) and rabbit anti-Calnexin (1:1,000; Immunoway, USA). The secondary antibody information is as follows: exosome validated goat anti-rabbit IgG (CD9, CD63 and TSG101; 1:5000; SBI) and BeriBlot for IP Detection Reagent (Calnexin, 1:2000; Abcam, UK).

### Assessment of acetylcholinesterase (AChE) activity

An Amplex^®^ Red Acetylcholine/Acetylcholinesterase Assay Kit (Thermo Fisher, USA) was used to determine the acetylcholinesterase (AChE) activity of the exosomes released into the supernatant. Briefly, 10 μL of exosome preparations from each group was first diluted to 100 μL in 1× reaction buffer and then added to a 96-well black flat-bottomed microplate (Greiner, Germany). Next, 100 μL of working solution containing Amplex Red reagent/HRP/choline oxidase/acetylcholine was pipetted into each microplate well containing the exosome samples and blank controls. The final reaction volume of 200 μL was incubated for 30 min at room temperature away from light. The fluorescence was measured in a fluorescence microplate reader (GloMax^®^-Multi Detection System, Promega, USA) using emission detection at 590 nm and was then corrected for background fluorescence by subtracting the value from the PBS control.

### Immunofluorescent staining

For intracellular distribution of CD63 in the hBMSCs after incubation for 48 h on different titanium topographies, cells were fixed with 4% paraformaldehyde for 10 min and blocked with PBS containing 0.1% Triton X-100 and 1% bovine serum albumin for 30 min. The cells on the titanium samples were first incubated with rabbit anti-CD63 (1:200; Immunoway) at 4 °C overnight and stained with goat anti-rabbit Alexa Fluor 488 IgG H&L antibody (1:500; Abcam) and DAPI-containing mounting medium (Beyotime). Images of the immunofluorescent staining were captured with a confocal laser scanning microscope (LSM780, Carl Zeiss Meditec AG, Germany).

### Quantitative real-time polymerase chain reaction (qRT-PCR)

Total RNA from the hBMSCs after incubation on the titanium sample for 48 h was extracted by a Direct-zol™ RNA MiniPrep Kit (Zymo Research, USA) following the manufacturer’s instructions. cDNA from each group was synthesized using a PrimeScript™ RT Master Mix Kit (Takara, Japan), followed by qRT-PCR on a Roche LightCycler^®^ 96 System using LightCycler^®^ 480 SYBR Green I Master (Roche, Switzerland). The primers for the target genes related to biogenesis and secretion of exosomes were as follows: RAB27A F: 5′-GATGCTTCTGGACCTGATAATGA-3′, R: 5′-CCACCTGAACTACTATGTCGCTT-3′; RAB27B F: 5′-GCCCTCACAGAGACACTAACACAG-3′, R: 5′-GTGAGGAGACCAAGAGAAGGCA-3′; SMPD3 F: 5′-ATGGACGTGGCCTATCACTGTT-3′, R: 5′-CTTGAGAAACAGAGCTCCCTTAGA-3′; and GAPDH F: 5′-GAACGGGAAGCTCACTGG-3′, R: 5′-GCCTGCTTCACCACCTTCT-3′.

### Western blot (WB)

The proteins from the hBMSCs after incubation on the titanium sample for 48 h were extracted by RIPA lysis buffer containing protease and phosphatase inhibitors (Beyotime) and quantified by a BCA Protein Assay Kit (Cowin Bio., PR China). Then, 20 μg of protein from each sample was loaded on a 12% precast mini polyacrylamide gel (GenScript, USA) and transferred to a PVDF membrane (Millipore, USA). The membrane was blocked with 5% skim milk (BD) dissolved in 1× Tris-buffered saline with Tween (TBST; Cowin Bio.) and probed with the following primary antibodies at 4 °C overnight: rabbit monoclonal anti-RAB27A (1:1000; Abcam), rabbit monoclonal anti-GAPDH (1:1000; CST, USA), rabbit polyclonal anti-RAB27B (1:1000; Abclonal, PR China), rabbit polyclonal anti-SMPD3 (1:1000; Abclonal) and rabbit polyclonal anti-SYTL4 (1:1000; Immunoway). The membrane was then incubated at room temperature for 1 h with goat anti-rabbit IgG (1:20,000; CST). The probed blots were developed using an ECL reagent (Millipore) and detected with a chemiluminescence imaging system (GeneGnome XRQ, USA).

### Animal experiment

The study protocol was approved by the Institutional Animal Care and Use Committee (IACUC) of Sun Yat-Sen University (Approval No. SYSU-IACUC-2020-000352). Thirty-two male SD rats (Laboratory Animal Center, Sun Yat-sen University, Guangzhou, China) weighing 200–250 g were used for this experiment. Anesthesia was induced by injecting pentobarbital sodium (2.5 mg/kg, intraperitoneally). In the left and right distal femoral bone planes, implant drilling holes with a diameter of 2.2 mm were prepared and randomly assigned to one of the above 4 titanium implant groups. The implants were directly placed and submerged for healing. Seven and 28 days after surgery, rats were sacrificed by injection of an overdose of pentobarbital sodium, and the distal femoral bone blocks containing the implants were collected and fixed in 10% formaldehyde for histological analysis.

### Immunohistochemistry and histological evaluation

The specimens euthanized after 7 days were decalcified in 10% ethylenediaminetetraacetic acid (EDTA) for 45 days and embedded in paraffin. The implants were unscrewed and 5‑μm‑thick sections were prepared and stained with hematoxylin and eosin (H&E). For immunohistochemistry, sections were incubated with rabbit anti‑CD63 antibody (1:100; Abclonal), rabbit anti‑periostin antibody (1:1000; Abcam) and rabbit anti‑CD11b antibody (1:4000; Abcam) overnight at 4 ℃ followed by incubation with a horseradish peroxidase (HRP)‑conjugated secondary antibody. Sections were examined with a digital pathology scanner (Aperio AT2; Leica Biosystems, USA) and evaluated by calculating the mean optical density (MOD) of CD63, periostin and CD11b expression using Image-Pro Plus 6.0 (Media Cybernetics, USA).

The specimens euthanized after 28 days were dehydrated in a gradient series of ethanol and embedded in polymethylmethacrylate. Sections with a final thickness of 50 μm were prepared using a saw (Leica SP1600; Leica Biosystems) and stained with methylene blue acid solution. Digital images at 25× magnification were acquired with an Axio Imager.Z2 (Zeiss) and analyzed for the bone-implant contact (BIC) ratio with Image-Pro Plus.

### Statistics

Each experiment was repeated three times. For the description of the data, all values are expressed as the mean ± standard deviation and were analyzed using the SPSS 25.0 software package (SPSS Inc., USA). One-way ANOVA was used to determine the level of significance. Differences among the groups were analyzed with the Student–Newman–Keuls correction. The level of significance was set at *p* < 0.05.

## Data Availability

All data generated or analyzed during this study are included in this published article.

## Abbreviations

AChE: Acetylcholinesterase
BMSCs: Bone marrow mesenchymal stem cells
EVs: Extracellular vesicles
qRT-PCR: Quantitative real-time polymerase chain reaction
SAH: Alkali-heat treatment
SAO: Anodic oxidation
SLM: Selective laser melting
SMPD3: Sphingomyelin phosphodiesterase 3
WB: Western blot
